# Rod‐Shaped Active Drug Particles Enable Efficient and Safe Gene Delivery

**DOI:** 10.1002/advs.201700324

**Published:** 2017-09-05

**Authors:** Xiaofei Xin, Xue Pei, Xin Yang, Yaqi Lv, Li Zhang, Wei He, Lifang Yin

**Affiliations:** ^1^ Department of Pharmaceutics, School of Pharmacy China Pharmaceutical University Nanjing 210009 P. R. China; ^2^ Key Laboratory of Druggability of Biopharmaceutics China Pharmaceutical University Nanjing 210009 P. R. China

**Keywords:** caveolar pathway, gene therapy, metastatic breast cancer, rod‐shaped drug particles, safety

## Abstract

Efficient microRNAs (miRNA) delivery into cells is a promising strategy for disease therapy, but is a major challenge because the available conventional nonviral vectors have significant drawbacks. In particular, after these vectors are entrapped in lysosomes, the escape efficiency of genes from lysosomes into the cytosol is less than 2%. Here, a novel approach for lethal‐7a (let‐7a) replacement therapy using rod‐shaped active pure drug nanoparticles (≈130 nm in length, PNPs) with a dramatically high drug‐loading of ≈300% as vectors is reported. Importantly, unlike other vectors, the developed PNPs/let‐7a complexes (≈178 nm, CNPs) can enter cells and bypass the lysosomal route to localize to the cytosol, achieving efficient intracellular delivery of let‐7a and a 50% reduction in expression of the target protein (KRAS). Also, CNPs prolong the *t*_1/2_ of blood circulation by ≈threefold and increase tumor accumulation by ≈1.5–2‐fold, resulting in significantly improved antitumor efficacies. Additionally, no damage to normal organs is observed following systemic injection of CNPs. In conclusion, rod‐shaped active PNPs enable efficient and safe delivery of miRNA with synergistic treatment for disease. This nanoplatform would also offer a viable strategy for the potent delivery of proteins and peptides in vitro and in vivo.

## 1 Introduction

MicroRNAs (miRNAs), a class of small noncoding RNAs, play a central role in various biological functions, such as cell proliferation, differentiation, apoptosis, and metabolism.^1^ Increasing evidence demonstrates that aberrant expression of miRNAs is closely related to human cancers; and thus, by regulating the expression of critical cancer‐related genes, miRNAs are capable of essentially inhibiting the tumor growth and angiogenesis.^2^ For example, lethal‐7a (let‐7a), one of the founding members in the miRNA family,^3^ is poorly expressed in human cancers including prostate, breast, and brain cancers, which is decreased by more than threefold in contrast to other healthy tissues.[[qv: 2b,4]] Accordingly, upregulation of let‐7a results in a marked promotion of apoptosis and inhibition of the proliferation and metastasis of cancer cell by silencing KRAS and high‐mobility group AT‐hook 2 (HMGA2).^5^ Therefore, miRNA replacement therapies can be employed as a promising treatment strategy if the miRNA can be delivered to cancer cells with high efficiency.[[qv: 5a,6]]

However, because of their high molecular weight (14 kDa) and highly negative charge, miRNA delivery is extremely difficult, exhibiting poor membrane penetration and thus limited cellular uptake, poor stability in vitro and in vivo, an inappropriate biodistribution, and off‐target effects, among others.^7^ Cationic vectors, which can complex with negatively charged genes through electrostatic interactions, in particular polyethyleneimines (PEI), synthetic poly(amidoamine) dendrimers (PAMAM), and cationic lipids,^8^ are most commonly used to overcome these barriers for miRNA delivery. Indeed, these vectors significantly improve the transfection efficiency. Nevertheless, the highly positive charge stems from the high density of amines on these vectors, which generates strong toxic effects; even at very low doses, the toxicity is still intense, hindering their further clinical application. Severe side effects, such as liver necrosis, shock response, and acute pulmonary embolism, have been observed following the intravenous injection of 1.5 × 10^−6^ m PEI (22 kDa) into mice with a body weight of 20 g.^9^ Second, after injection, these conventional cationic vectors bind to serum protein and thus are rapidly removed from the blood, with a circulation time less than 10 min,^10^ thus preventing localization to the desired tissues. Additionally, these vector/gene complexes tend to accumulate in lysosomes followed by internalization, and subsequently the obtained gene escapes from lysosomes by taking advantage of the “proton sponge effect” into the cytosol of target cells, where they associate with the target gene to exert their silencing function.^11^ Unfortunately, the escape efficiency of genes from lysosomes into the cytosol is extremely low at only 1–2%, and it only occurs in a short limited time‐period when the vectors are located in a specific compartment that shares both early and late endosomal characteristics.^12^ Critically, during this trafficking process, significant lysosomal degradation of the gene by digestive enzymes and acidic conditions with a pH less than 4.5 occurs in lysosomes,^13^ thereby greatly diminishing the therapeutic efficacy. Thus, lysosomal decomposition is a critical obstacle for intracellular miRNA delivery.^14^ Consequently, new vectors with efficient delivery of miRNAs and perfect biocompatibility are highly desirable.

**Scheme 1:**
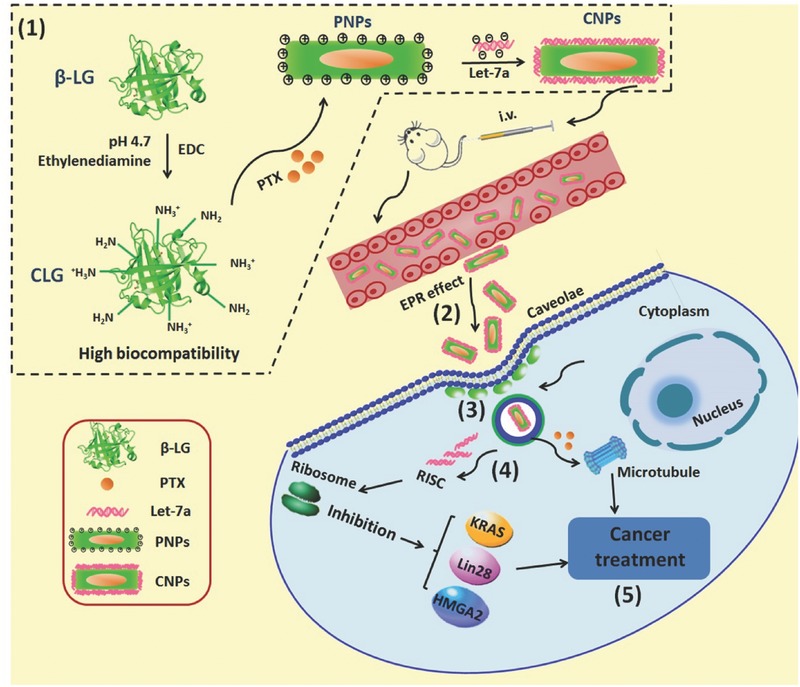
Illustration of (1) preparation of CNPs and purpose active‐procedure for cancer treatment: after intravenous injection, CNPs (2) accumulate in tumor via an EPR effect, (3) enter the cancer cells through caveolae endocytosis without entrapment in the endosomal–lysosomal system, and (4) directly deliver miRNA to the cytosol, thereby protecting miRNA from enzymatic degradation, and finally (5) achieve potent cancer treatment with miRNA or the combination of miRNA and PTX.

Caveolae‐mediated endocytosis has been an area of intense focus owing to the absence of entrapment in the acidic and digestive conditions of lysosomes, rendering a greater amount of active drug delivery into the target cell cytosol with high efficiency and thus making it a promising route for the subcellular delivery of biomacromolecule drugs, such as proteins and genes.[[qv: 14c,e,15]] In a previous report,^16^ we demonstrated that rod‐shaped pure drug nanoparticles (PNPs) of paclitaxel (PTX) with a length of 160 nm and an aspect ratio of five entered cancer cells via caveolae. Additionally, these rod‐shaped PNPs showed significantly enhanced in vivo performance resulting in a dramatically prolonged half‐life in blood and profound tumor penetration with a 100% penetration ratio.^16^, ^17^ Because of these excellent features, it was hypothesized that these PNPs were capable of serving as promising vectors for miRNA delivery. The purpose of the present paper is depicted in, **Scheme 1**: following intravenous injection, cationic PNPs/let‐7a complexes (CNPs) will accumulate in the tumor via an enhanced permeability and retention (EPR) effect and then enter the cancer cells through the caveolar pathway without entrapment in lysosomes, thereby delivering the miRNA into the cytosol without lysosomal degradation. To obtain proof‐of‐concept, various in vitro and in vivo experiments were conducted. The potential benefits of the present miRNA delivery system include (i) dramatically prolonged blood circulation and resultant accumulation in the tumor, (ii) achievement of cellular entry via a nonlysosomal route and protection of miRNA against decomposition by enzymatic and acidic conditions, and (iii) combination use of drugs and miRNA for disease therapy with low toxicity. This work provides a valuable approach for efficient and safe miRNA delivery, as well as protein therapy.

## 2 Results

### 2.1 Preparation and Characterization of PNPs and CNPs

PNPs were prepared by a precipitation–ultrasonication method^18^ using cationic β‐lactoglobulin (CLG) as a stabilizer that functioned to prevent PNPs from aggregating by coating the drug particles and allowing the PNPs to achieve a positive surface charge to condense the gene. To verify the stabilization effect from the interplay between the drug particles in PNPs and CLG, fluorescence and far‐UV circular dichroism (CD) spectra were examined. After selective excitation of tryptophan (Trp) residues located in the protein' structure, CLG would generate maximum fluorescence at ≈340 nm, yet the fluorescence would be altered once the local environment of Trp was disturbed due to the conformational alternation of the protein. It was assumed that if CLG was coated onto the drug particles, the protein would suffer from conformational changes; therefore, the conformational analysis by fluorescence or CD spectra could, in turn, confirm the interaction between the drug particles and protein. Despite the difference in drug‐loading, the addition of drug particles resulted in significant fluorescence quenching compared with CLG and consequently demonstrated a transition of the Trp residues to a less hydrophobic environment and conformational changes of the protein, thus validating an interaction between the protein and the drug particles (Figure S1A, Supporting Information). Indeed, an increase in fluorescence also resulted from an increase in drug‐loading. This phenomenon was probably due to the increased hydrophobic area from the greater number of drug particles benefiting protein absorption, consequently compensating for the exposure of the Trp residues to a less hydrophobic environment. The conformational change in the secondary structure of CLG coated on the drug particles was further investigated by the CD spectrum. CLG exhibited a typical far‐UV CD spectrum featured by a β‐sheet structure and an α‐helix with a wide negative minimum at ≈218 nm (Figure S1B, Supporting Information). After mixing the drug particles with CLG, the negative minimum, indicative of α‐helix content, was markedly reduced, demonstrating a trend in which the negative minimum declined with an increase in drug‐loading and thus demonstrating alterations in the secondary structure of CLG. Collectively, these results indicated a robust interaction between CLG and the drug particles in PNPs and a profound stabilization effect of CLG on the hydrophobic drug particles.

**Figure 1:**
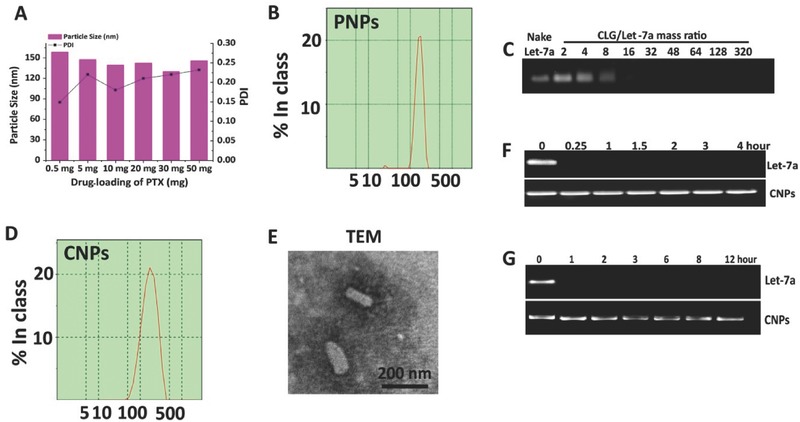
Characterization of PNPs and CNPs. A) The impact of the drug‐loading of PTX from 0.5 mg (5%) to 50 mg (500%) on the particle size in length of PNPs. Size distribution of B) PNPs from optimized formulation and D) CNPs. C) Gel electrophoresis study of CNPs at CLG/let‐7a mass ratios ranging from 2 to 320. E) TEM image of CNPs. Protection of let‐7a from degradation by F) RNase or G) serum evaluated by agarose gel electrophoresis. Let‐7a alone or CNPs were incubated with 50% serum solution or 10 µg mL^−1^ RNase A at 37 °C for different durations.

First, the drug‐loading of PTX on the particle size in length of PNPs was studied. The particle size of PNPs showed a reducing trend with increasing drug‐loading from 5% (0.5 mg) to 500% (50 mg) (**Figure** **1**A), along with all sizes less than 160 nm. Plus, polydispersity index (PDI) values in these formulations were smaller than 0.3 and, therefore, indicated a narrow size distribution. These results demonstrated the extremely high drug‐loading in PNPs due to the excellent stabilization effect of CLG on the drug particles in PNPs confirmed by fluorescence and far‐UV CD spectra (see Figure S1A,B in the Supporting Information). The smallest size of PNPs, ≈130 nm with a PDI of 0.23 and surface charge of 21.38 mV, was exhibited in the formulation with drug‐loading of 300% (30 mg) (Figure 1B).

Next, to optimize the formulation of CNPs, PNPs were mixed with let‐7a at mass ratios of CLG/let‐7a that were changed from 2 to 320 and incubated at room temperature for 30 min, followed by a gel electrophoresis assay. As the CLG/let‐7a ratio was increased to 16, the migration of let‐7a on the gel was completely retarded (Figure 1C), and thus this ratio was used in the optimized formulation of CNPs. The CNPs had a size of 178.0 nm in length with a PDI of 0.223, indicating homogeneous dispersion (Figure 1D), and possessed a potential of 20.36 mV. Tramsmission electron microscopy (TEM) examination of the CNPs revealed rod‐like particles that were ≈160 nm in length, consistent with the dynmic light scattering (DLS) results (Figure 1E).

### 2.2 In Vitro Stability and Drug Release

To assess the ability of CNPs to protect let‐7a from degradation, the stability of let‐7a in RNase solution and 10% fetal bovine serum (FBS) was studied. As shown in Figure 1F,G, the band of naked let‐7a entirely disappeared upon exposure to RNase or serum for 0.25 h or 1 h; by contrast, the band of let‐7a in CNPs was clearly visible after incubation with RNase for 4 h or with FBS for 12 h. These results implied that CNPs were capable of protecting let‐7a against decomposition by serum and RNase.

The release profiles of PTX from the CNPs, PNPs or Taxol (a marketed formulation) were studied using dialysis method in media at pH 7.4, 6.8 or 5.0. In general, the release from Taxol was faster than that of PNPs or CNPs because PTX existed as a free drug in Taxol but as solid particles in PNPs or CNPs. Importantly, the percent of PTX release from PNPs or CNPs was less than 10% at 3 h and from 40 to 60% at 48 h (Figure S1C–E, Supporting Information), demonstrating that these two nanoparticles were stable under physiological conditions within a short period, yet would ultimately disassemble and release their active components.

### 2.3 Caveolae‐Mediated Cellular Uptake and Colocalization of CNPs with Caveolae

**Figure 2:**
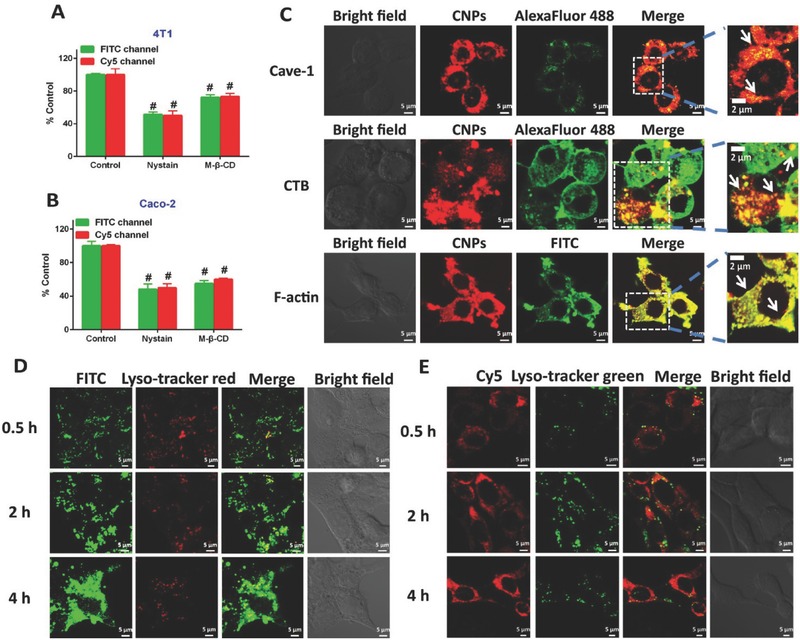
Caveolae‐mediated uptake and trafficking. Cellular uptake of dual‐labeled CNPs in A) 4T1 or B) Caco‐2 cells pretreated with caveolae inhibitors, nystatin (10 × 10^−6^ m) or M‐CD (2.5 × 10^−3^ m), for 0.5 h at 37 °C. Dual‐labeled CNPs were incubated with the pretreated cells at 37 °C for 4 h at 7.5 µg mL^−1^ FITC or 100 × 10^−9^ m Cy5. The fluorescence intensity was assayed by flow cytometry. ^#^ *P* < 0.001 versus control (*n* = 5). C) Colocalization of Cy5‐CNPs with FITC‐labeled F‐actin (green), Alexa Fluor 488‐labeled CTB (green), and Alexa Fluor 488‐labeled Cave‐1 (green). Yellow spots display the colocalization of CNPs with F‐actin, CTB, or Cave‐1. The 4T1 cells were incubated with CNPs for 4 h at 37 °C. The location of the FITC‐CNPs (10 µg mL^−1^, D) or Cy5‐CNPs (100 × 10^−9^ m let‐7a, E) in lysosomes of 4T1 cells after incubation for 0.5, 2, and 4 h at 37 °C, respectively. Yellow spots indicate the colocalization of CNPs with lysosomes. The scale bar is 5 µm.

Nystatin and methyl‐β‐cyclodextrin (M‐CD) are two typical inhibitors for blocking caveolae internalization.^15^, ^19^ Therefore, the uptake in cells preincubated with these inhibitors was investigated. The uptake of dual‐labeled CNPs in 4T1 cells was decreased by 40% and 30% after the caveolar pathway was suppressed with nystatin or M‐CD (**Figure** **2**A), despite the difference in use of fluorescence probes. Caco‐2 cells, in which caveolae constituted 50% cell surface area,^19^ were further selected to validate the role of the caveolar pathway in CNPs uptake. Again, the uptake of CNPs by Caco‐2 cells declined to ≈52% as caveolae endocytosis was inhibited by nystatin or M‐CD (Figure 2B). These data demonstrated that caveolar endocytosis played an essential role in cellular uptake of CNPs.

To study the colocalization of the CNPs with caveolae, Cave‐1, which is a specific protein for the formation of caveolae, was stained with Alexa Fluor 488 (green) prior to uptake by 4T1 cells. As shown in Figure 2C, yellow spots were observed in the merged image and therefore confirmed the colocalization of caveolae with Cy5‐CNPs. Apart from Cave‐1, the actin cytoskeleton and cholera toxin subunit B (CTB) were also involved in caveolar trafficking. Thus, the colocalization of Cy5‐CNPs with these two markers was further studied. Indeed, after colocalizing Cy5‐CNPs (red) with actin or CTB (green) stained green by fluorescein isothiocyanate isomer I (FITC) phalloidine and Alexa Fluor 488‐CTB, respectively, strong yellow fluorescence was observed in the merged pictures, implying the involvement of caveolar trafficking (Figure 2C). Generally, caveolar internalization allowed the nanoparticles to enter the cells bypassing the lysosomes. Accordingly, the intracellular trafficking of FITC‐CNPs or Cy5‐CNPs in lysosomes of 4T1 or Caco‐2 cells was studied. Little yellow fluorescence was visualized in the overlaid images of lysosomes stained red or green with FITC‐CNPs (green) or Cy5‐CNPs (red) at 0.5, 2, and 4 h postincubation (Figure 2D,E), indicating that the CNPs entered the cells without entrapment in lysosomes. Additionally, the integrity of CNPs during cellular trafficking was investigated after incubation of dual‐labeled CNPs with 4T1 or Caco‐2 cells for 4 h and examined by confocal laser scanning microscopy (CLSM). Robust yellow fluorescence shown in the merged images indicated the integrity of CNPs in cells and codelivery of PTX and let‐7a (Figure S2, Supporting Information). Examination of live‐cell imaging exhibited that cytosolic delivery of Cy5‐CNPs was time‐dependent, with the strongest red fluorescence in the cytoplasm occurring at ≈23.5 min (Figure S3, Supporting Information).

### 2.4 Transcytosis and Penetration of Tumor Spheroids

**Figure 3:**
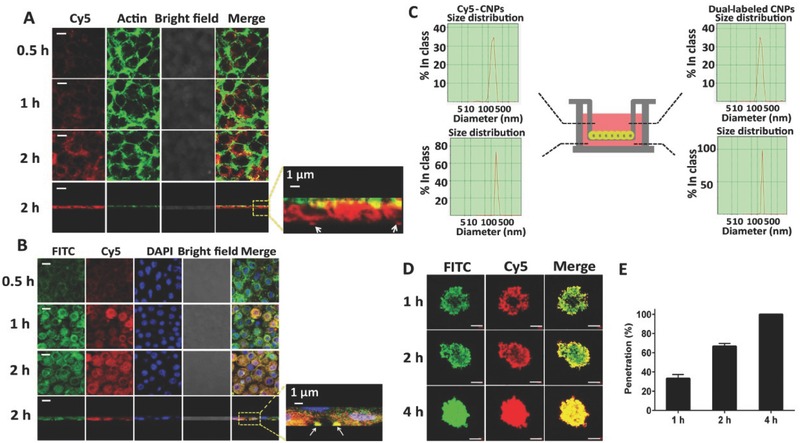
Transcytosis and penetration. A) The colocalization of Cy5‐CNPs (red) with FITC labeled F‐actin (green) in the Caco‐2 cell monolayer after incubation at 37 °C for various durations at a dose of 100 × 10^−9^ m Cy5‐let‐7a. The image was obtained from the *X*–*Y* plane at a depth of 15 µm along the *Z*‐axis. Yellow or orange spots indicate the colocalization of CNPs with actin. The scale bar is 5 µm. B) The colocalization of dual‐labeled CNPs with Caco‐2 cell monolayer after incubation at 37 °C for various durations at a dose of FITC 10 µg mL^−1^ or Cy5‐let‐7a 100 × 10^−9^ m. The nuclei (blue) were stained with DAPI. The depth for CLSM observation was 10 µm along the *Z*‐axis. The scale bar is 5 µm. Yellow or orange spots demonstrate the integrity of CNPs during the transcytosis process. A/B: The fourth line panels are the *X*–*Z* vertical confocal images of the Caco‐2 cell monolayer at 2 h. White arrows indicate the CNPs crossed the Caco‐2 cell monolayer. The scale bar is 5 µm. C) DLS results of CNPs in the apical or the basolateral side under the condition of the Caco‐2 cells monolayer incubated with Cy5‐CNPs or dual‐labeled CNPs at 37 °C for 2 h. D) In vitro penetration of dual‐labeled CNPs into the 3D multicellular 4T1 tumor spheroids after incubation at 37 °C for different times. *X*–*Y*–*Z* images of CLSM were obtained at a depth of 19 µm. The scale bar is 50 µm. E) The penetration percent of CNPs at 4T1 tumor spheroids at 1, 2, and 4 h.

Penetration of a drug into tumor is a critical factor for tumor therapy; hence, it is necessary to test transcytosis and tumor penetration. Initially, the transcellular transport of CNPs across the Caco‐2 cell monolayer was conducted via CLSM observation in X‐Y‐Z scanning mode after incubation with Cy5‐CNPs or dual‐labeled CNPs. After incubation for 2 h with Cy5‐CNPs, intense red fluorescence on the basal side and yellow fluorescence distributed in the monolayer in the merged image were observed (enlarged view, **Figure** **3**A and Figure S4A, Supporting Information). In the *X*–*Y* plane of the Caco‐2 cell monolayer along the *Z*‐axis at a depth distance of 10 µm, the colocalization of Cy5‐CNPs with actin was confirmed (Figure 3A). Additionally, yellow fluorescence obtained by overlaying the green FITC fluorescence and Cy5 red fluorescence from the dual‐labeled CNPs appeared in the merged images at 2 h postcoincubation of dual‐labeled CNPs with the cell monolayer with blue‐stained nuclei (enlarged view, Figure 3B and Figure S4B, Supporting Information). Similar results were obtained from the CLSM examination in an X–Y model (Figure 3B). The size of transported PTX‐Ns in basolateral media was similar to that in apical media (Figure 3C). Collectively, these results indicated that the CNPs penetrated through the Caco‐2 cell monolayer and achieved transcytosis without disassembly.

Next, the penetration of dual‐labeled CNPs into multicellular spheroids was investigated. As the incubation time increased, spheroids treated with dual‐labeled CNPs showed green or red fluorescence distributed from the periphery to the central regions of the spheroids, with the strongest fluorescence accumulating throughout the entire spheroid at 4 h; yellow fluorescence in the merged images at each time points was also observed, indicating intact CNPs during the penetration process (Figure 3D). A quantitative assay was conducted to determine the penetration ability by calculating the penetration ratio (PR) of the penetration depth and spheroid radius (150 µm). The PR values for the CNPs were ≈100%, 67%, and 33% at 4, 2, and 1 h, respectively (Figure 3E). These results indicated that CNPs possessed an excellent ability to penetrate the tumor.

### 2.5 Transfection Efficiency and Migration Inhibition

**Figure 4:**
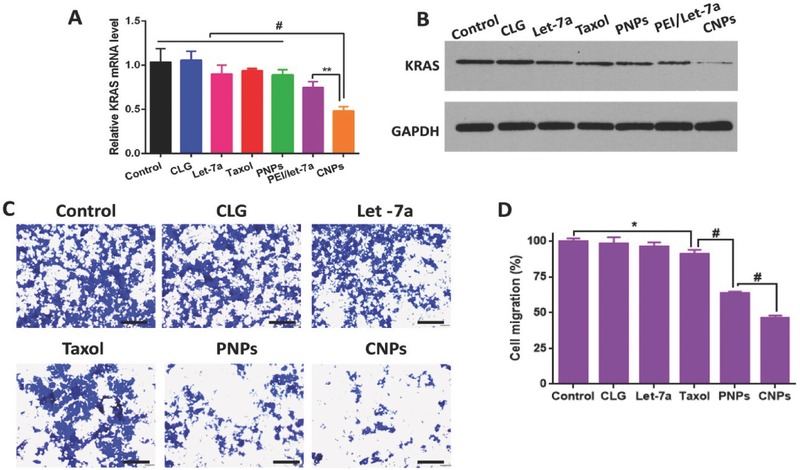
In vitro transfection and migration inhibition. A) Expression of KRAS detected by real‐time PCR. Cells without treatment represent the control, and the related data were set at 1.0 (*n* = 3, ^**^ *P* < 0.01 vs control). B) KRAS expression examined by western blot analysis. GAPDH was utilized as an internal control to normalize protein expression. Cells without treatment served as the control. C) Migration inhibition of 4T1 cells investigated by a transwell chamber assay. Blue spots demonstrate the migrated cells. The control group was the normal cells without treatment. The scale bar is 100 µm. D) Quantitative analysis of cell migration (*n* = 3, *P < 0.05 and ^#^ *P* < 0.001 vs control).

By directly down‐regulating the KRAS oncogene through their 3′UTR,′ let‐7a inhibited the tumor growth and migration of cancer cells.^6^ The expression of the targeted protein, KRAS, in transfected 4T1 cells was studied by real‐time PCR and western blot analysis. Compared with other controls, a 50% reduction in KRAS expression was obtained in cells treated with CNPs (**Figure** **4**A); the western blot assay also indicated that the band signal of KRAS from CNPs was weakest among these groups (Figure 4B). These results indicated that CNPs‐mediated intracellular delivery of let‐7a effectively suppressed the expression of KRAS and thus generated a high transfection efficiency.

The migration of cancer cells was a predominant element leading to cancer recurrence.^20^ Compared with the saline group, CLG, Taxol (free drug formulation), and naked let‐7a did not suppress cell migration (Figure 4C,D). The weak inhibition of naked let‐7a on migration was ascribed to its poor membrane penetration. In contrast, PNPs and CNPs caused 62% and 49% cell migration, with the latter having a more profound migration inhibition effect (*P* < 0.05), thereby demonstrating the synergistic effect of PTX and let‐7a. Importantly, CNPs dramatically inhibited the migration in contrast with naked let‐7a, indicative of efficient intracellular delivery of let‐7a to cancer cells.

### 2.6 Cytotoxicity and Cellular Apoptosis

**Figure 5:**
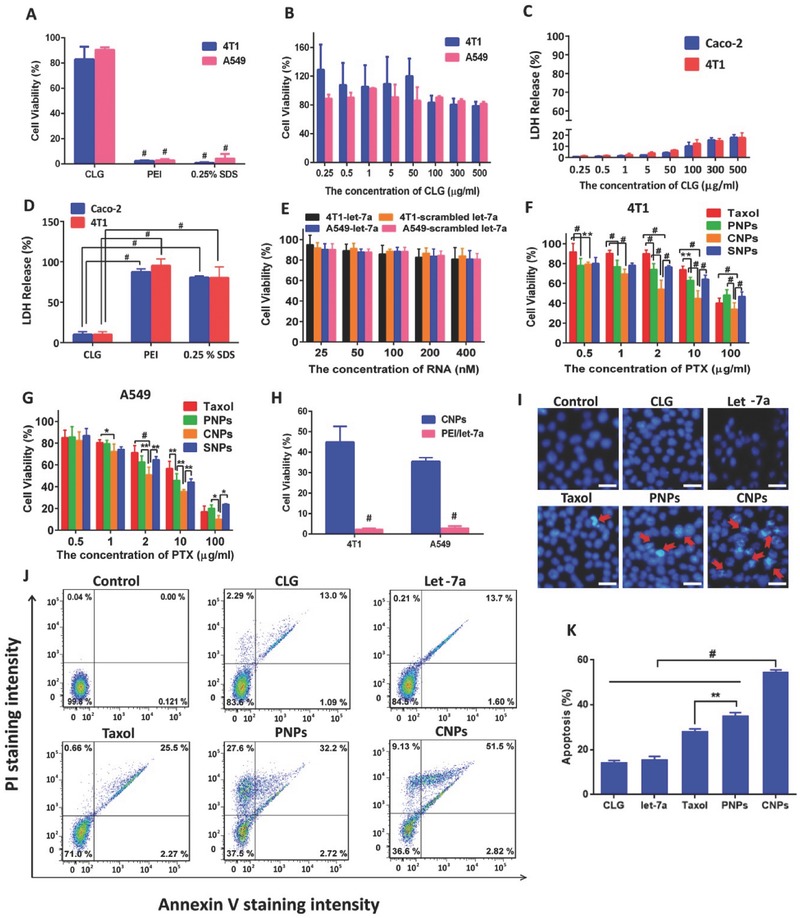
Cytotoxicity and apoptosis. A) Comparison of the cell viability among CLG (100 µg mL^−1^), PEI (100 µg mL^−1^), and SDS (0.25%, w/v). These materials were cultured with cells for 48 h at 37 °C (*n* = 5, ^#^ *P* < 0.001). B) Cell viability after incubation with CLG in 4T1 and A549 for 48 h at 37 °C (*n* = 5). The dose of CLG was ranged from 0.25–500 µg mL^−1^. C) LDH release from Caco‐2 and 4T1 cells incubated with different concentrations of CLG for 4 h at 37 °C. D) LDH release from Caco‐2 and 4T1 cells incubated with 100 µg mL^−1^ of CLG, 100 µg mL^−1^ of PEI and 0.25% SDS for 4 h at 37 °C (*n* = 5, ^#^ *P* < 0.001). E) Cell viability after incubation with naked let‐7a or scrambled let‐7a in 4T1 and A549 for 48 h at 37 °C (*n* = 5). The dose of let‐7a was from 20 × 10^−9^–400 × 10^−9^ m. Cell viability after incubation with Taxol, PNPs, SNPs or CNPs in F) 4T1 and G) A549 cells for 48 h at 37 °C (*n* = 5). The dose of PTX was ranged from 0.5–100 µg mL^−1^. H) Comparison of the cell viability among CNPs (CLG 100 µg mL^−1^, PTX 10 µg mL^−1^, and let‐7a 100 × 10^−9^ m), PEI/let‐7a (PEI 100 µg mL^−1^ and let‐7a 100 × 10^−9‐^ m) (*n* = 5, ^#^ *P* < 0.001). These formulations were incubated with cells for 48 h at 37 °C. I) Cell apoptosis induction and morphological changes in 4T1 cells produced by CLG, let‐7a, Taxol, PNPs, and CNPs containing a fixed PTX concentration of 10 µg mL^−1^ and let‐7a dose of 100 × 10^−9^ m in free let‐7a and CNPs for 48 h. Red arrows show cell apoptosis. The scale bar is 5 µm. J) Flow cytometry analysis of apoptosis induced by CLG, let‐7a, Taxol, PNPs, and CNPs containing a fixed PTX concentration of 10 µg mL^−1^ and let‐7a 100 × 10^−9^ m in free let‐7a and CNPs at 37 °C for 48 h in 4T1 cells. Cells were stained with FITC‐Annexin V and PI. K) Percentage of apoptotic cells determined by flow cytometry (*n* = 3, ^#^ *P* < 0.001 and ^**^ *P* < 0.01).

The cytotoxicity of vectors without drug was evaluated in A549 and 4T1 cells. The positive control (0.25% sodium dodecyl sulfate, SDS) and typical cationic polymer PEI had remarkable cytotoxicity with cell viability less than 10% after incubation with PEI for 48 h at a concentration of 100 µg mL^−1^ (**Figure** **5**A). In contrast, the CLG almost did not generate toxicity to the cancer cells, even at a concentration up to 500 µg mL^−1^ (Figure 5B). The effects of CLG, PEI, and SDS on cell membrane integrity were further investigated by the lactate dehydrogenase (LDH) release assay, in which LDH release occurs as the membrane is damaged.^21^ Compared with 100 µg mL^−1^ PEI and 0.25% SDS, a negative influence of CLG on the integrity of the cell membrane of 4T1 or Caco‐2 cells was observed at the tested concentrations ranging from 0.25 to 500 µg mL^−1^ (Figure 5C,D). These results demonstrated that CLG possessed little cytotoxicity against A549, 4T1 or Caco‐2 cells and thereby supported its profound biocompatibility.

The cytotoxicity of PNPs, SNPs (scrambled let‐7a/PNPs complexes), and CNPs against 4T1 and A549 cells was further tested, using naked let‐7a and free PTX (Taxol) as controls. Both naked and scrambled let‐7a displayed no cytotoxicity due to its poor membrane penetration (Figure 5E). PNPs, SNPs, and CNPs exhibited dose‐dependent cytotoxicity at PTX concentrations of 0.5–100 µg mL^−1^ (Figure 5F,G). PNPs promoted cytotoxicity compared with Taxol due to their efficient uptake by the cancer cells. SNPs and PNPs showed similar toxicity to cancer cells, while the cell viability from CNPs was significantly less than that from PNPs at a PTX concentration greater than 2 µg mL^−1^, demonstrating the supplemental effect of let‐7a and synergy between PTX and let‐7a. Additionally, the comparative cytotoxicity between CNPs and PEI/let‐7a in 4T1 and A549 cells was examined (Figure 5H). Both CNPs and PEI/let‐7a had significant toxicity to 4T1 or A549 cells at a let‐7a concentration of 100 × 10^−9^ m, while the latter almost killed all the cells, probably induced by the strong toxicity of PEI. This result confirmed the safety of CNPs.

Cell apoptosis was first evaluated by staining the nuclei after incubation and observation by fluorescence microcopy. Apoptosis was featured as a change in nuclear change characterized by chromatin condensation, fragmentation, and apoptotic body formation. Compared with the nuclei treated with PBS or CLG, significant chromatin condensation from PNPs and CNPs was displayed, and the condensation from CNPs was more profound; while the cells treated with naked let‐7a or Taxol exhibited little condensation (Figure 5I). Quantitative analysis by flow cytometry (FCM) using Annexin V‐FITC/PI apoptosis detection kits also indicated that CNPs with a total percent of apoptotic 4T1 cells of 54.3% displayed significantly higher apoptotic activity in contrast to PNPs (34.9%) or other controls (Figure 5J,K). Accordingly, CNPs were able to induce cell apoptosis with high efficiency.

### 2.7 Blood Circulation, Ex Vivo Imaging, and Biodistribution

**Figure 6:**
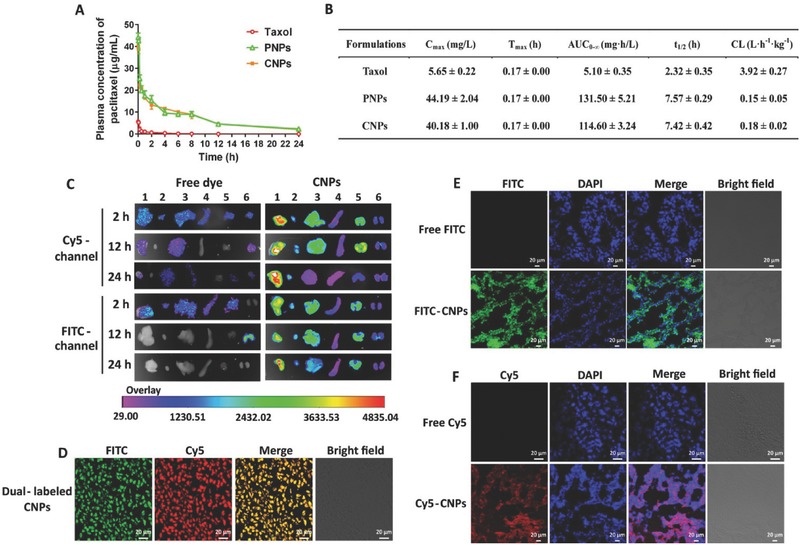
Blood circulation, biodistribution, and tumor accumulation. A) Plasma concentration–time curves and B) comparative pharmacokinetic parameters of Taxol, PNPs, and CNPs after intravenous administration into rats at a PTX dose of 10 mg kg^−1^ (*n* = 4). *C*_max_, maximum plasma concentration; AUC, area under the concentration curve; *t*_max_, time to reach *C*_max_; *t*_1/2_, biological half‐life; CL, clearance. C) Ex vivo fluorescence images of tissues, including tumor (1), heart (2), liver (3), spleen (4), lung (5), and kidneys (6) collected at 2, 12, and 24 h postinjection of dual‐labeled CNPs. Free FITC and Cy5 were used as controls. Biodistribution of CNPs in tumors harvested from 4T1 tumor‐bearing mice injected with D) dual‐labeled CNPs, E) FITC‐CNPs, or F) Cy5‐CNPs at PTX dosage of 10 mg kg^−1^ and let‐7a dose of 1 mg kg^−1^ for 2 h. The frozen tumor sections were observed using CLSM. The nuclei were stained with DAPI. The scale bar is 20 µm.

The duration of blood circulation and the plasma concentration are two critical factors for nanomedicine to obtain the target tissue accumulation after intravenous injection.^22^ Thus, we investigated the pharmacokinetics of these vectors in rats. Taxol exhibited low plasma levels of PTX postinjection, followed by a rapid decrease to undetectable levels at 4 h; whereas the plasma levels of the PNPs and CNPs were markedly higher than Taxol, demonstrating an ≈sevenfold increase (**Figure** **6**A,B). Crucially, the *t*_1/2_ for blood circulation from PNPs or CNPs was extended by ≈threefold compared with Taxol (Figure 6B), the area under the curve (AUC) increased by more than 22‐fold and clearance (CL), a measurement of the volume of plasma from which a drug is completely removed per unit time, declined by ≈25‐fold postinjection of PNPs or CNPs. Our previous work showed that similar rod‐like particles with a negative instead of a positive charge had an extremely prolonged blood circulation.^16^, ^17^ These results demonstrated that the profound pharmacokinetics of these rod‐like particles was based on their shape but not their surface. Collectively, both PNPs and CNPs exhibited significant improvements in pharmacokinetics, including increased AUC, prolonged blood circulation and decreased blood clearance, thereby benefiting their accumulation in tumors via the EPR effect.

Next, ex vivo imaging was performed to study the accumulation of dual‐labeled CNPs in tissues of 4T1 tumor‐bearing mice postinjection. Dual‐labeled CNPs displayed strong FITC or Cy5 fluorescence intensity in the tumor at 2 h postinjection compared with free dyes; and even at 24 h, the fluorescence signal was not weakened (Figure 6C). Interestingly, among these tissues, the fluorescence signal was most robust in the tumor, followed by liver and lung (the fluorescence intensity of the tumor was ≈1.5–2‐fold greater than the other two organs at 2 h postinjection). These results suggested that CNPs were capable of effectively accumulating in the tumor, consistent with our previous report that rod‐like drug particles are well distributed in tumors.^16^ In particular, at 24 h after dosing, significant reduction of CNPs accumulation in liver and lung was observed, suggesting that CNPs were able to be cleared from the body without causing potential damage to these two normal organs. Furthermore, CLSM observation of the sectioned tumor tissues collected at 2 h postinjection of dual‐labeled CNPs exhibited yellow fluorescence in the merged image (Figure 6D), thereby indicating that the CNPs accumulated in the tumor was intact. The distribution of CNPs in the tumor was further studied by CLSM examination of frozen tumor sections after injection of FITC‐CNPs or Cy5‐CNPs. Green (FITC) or red (Cy5) fluorescence distributed in whole tumor sections was visualized in the merged images (Figure 6E,F) and demonstrated that CNPs penetrated the tumor, which was ascribed to their transcytosis ability (Figure 3). Altogether, these results suggested that CNPs possessed efficient tumor targeting ability.

### 2.8 Therapeutic Efficacy

**Figure 7:**
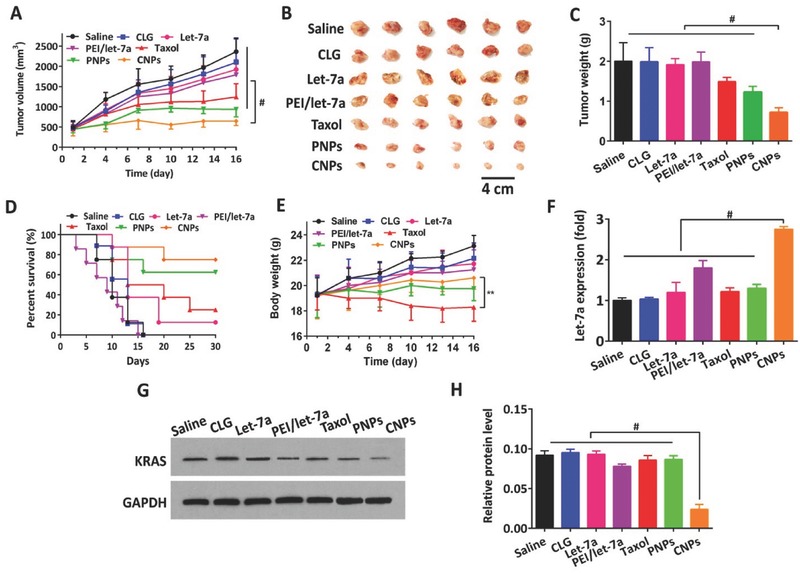
Antitumor activities. Saline, CLG, Taxol, PNPs, and CNPs were administered to 4T1 tumor‐bearing mice via tail vein injections every 3 d at a PTX dose of 10 mg kg^−1^ and let‐7a dose of 1 mg kg^−1^, according to the animal's body weight. The injection volume was 0.2 mL. A) Tumor growth curves. B) Image of representative tumors collected from mice at the end of the experiment. C) Tumor weight variation (*n* = 12, ^#^ *P* < 0.001). D) Survival curves (*n* = 12, ^**^ *P* < 0.01, ^#^ *P* < 0.001), and E) body weight change curves. A comparison of the final tumor volume or body weight between groups was performed at day 16. F) Intratumoral delivery of let‐7a examined by real‐time PCR (*n* = 3, ^#^ *P* < 0.001). G) KRAS expression in tumor from mice detected by western blot analysis. H) Quantitative analysis of KRAS expression in tumors from mice determined by the BCA protein assay (*n* = 3, ^#^ *P* < 0.001).

The antitumor efficacies of CLG (vectors without drug), Taxol, naked let‐7a, PEI/let‐7a, PNPs, and CNPs were evaluated with respect to tumor volume, tumor weight, body weight, and survival rate in 4T1 tumor‐bearing BALB/c mice, using saline and Taxol (free drug) as the negative and positive controls, respectively. Taxol, PNPs, and CNPs suppressed tumor growth significantly at day 16 after administration; indeed, the greatest inhibition was obtained with CNPs, for which tumor growth was completely terminated following two treatments at day 4. Subsequently, the tumor showed no additional growth (**Figure** **7**A,B). Importantly, the tumor volume of CNPs was approximately onefold less than that of PNPs without let‐7a at 16 d post‐treatment, indicating the efficient delivery of let‐7a to the tumor. The weight measurement of isolated tumor collected at the end of experiment further demonstrated that CNPs inhibited the tumor growth with the highest efficiency (Figure 7C), with values 1.66‐ and 2.52‐fold less that PNPs and Taxol, respectively, corresponding to the tumor volume measurements. We also determined the survival rate of mice at day 30 after injection. The survival rate for CNPs was 75.0%, which was significantly greater than saline (0%), CLG (0%), Taxol (25%), and PNPs (62.5%) (Figure 7D). These results were partly related to the reduced toxicity from CNPs, as evidenced by that small amount of body‐weight loss among the drug‐containing vectors except Taxol (Figure 7E).

To investigate the intratumoral delivery of let‐7a with CNPs, real‐time PCR was conducted on the tumor at the end of treatment. The level of let‐7a in tumor post‐treated with CNPs was markedly higher than that from other controls, along with an ≈1.5‐fold increase (Figure 7F). Moreover, let‐7a exerted its antitumor activity by silencing KRAS mRNA.[[qv: 2b]] To ascertain whether the expression of KRAS mRNA was reduced in tumors for let‐7a treatment, western blotting and BCA protein assays were applied. Western blotting showed that the expression of KRAS was markedly downregulated following CNPs treatment (Figure 7G) compared with other controls, and further quantitative analysis demonstrated that KRAS was reduced threefold post‐treatment with CNPs (Figure 7H).

**Figure 8:**
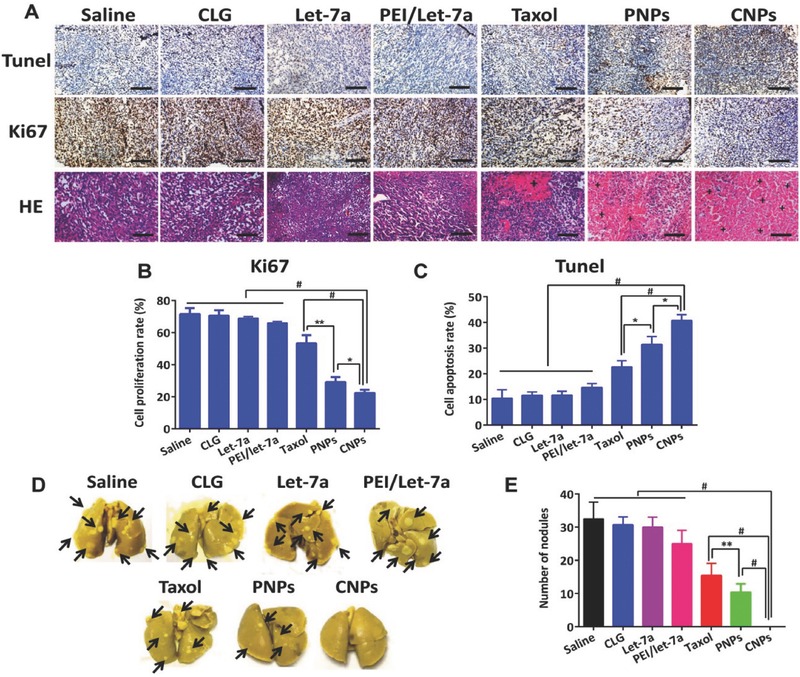
Apoptosis and proliferation in tumor and lung metastasis. A) TUNEL, Ki67, and H&E staining analysis of tumor. The brown‐staining cells represent the positive cells in the TUNEL and Ki67 assay. Nuclei are stained blue while extracellular matrix and cytoplasm are stained red in the H&E analysis. “+” represents the positive area. The scale bar is 20 µm. Quantitative analysis of B) cell proliferation and C) apoptosis (*n* = 3, *P < 0.05). D) The images show cancer metastasis in lungs excised from the mice. Black arrows indicated metastatic nodules in lungs. E) Quantitative analysis of nodules in lung (*n* = 6, **P < 0.05, ^#^ *P* < 0.01).

To further determine the cell apoptosis and lowest cell proliferation in the tumor, terminal deoxynucleotidyl transferase dUTP nick end labeling (TUNEL) and Ki67 analysis were performed on the sectioned tumor after the treatment was completed. CNPs exhibited the highest level of stimulated cell apoptosis (43%) and the lowest cell proliferation (19%), respectively, following by PNPs at 31% and 30% and Taxol at 21% and 53% for cell apoptosis and cell proliferation, respectively (**Figure** **8**A–C). The hematoxylin and eosin (H&E) staining assay further illustrated that the fewest cell numbers in the tumor tissues were observed after treatment with CNPs compared with the other groups (Figure 8A).

The 4T1 tumor is a highly metastasis breast cancer, predominantly metastasizing to the lung and secondarily to the liver.^20^, ^23^ Accordingly, metastasis of 4T1 tumor to lung in 4T1 tumor‐bearing BALB/c mice was investigated at day 16 postadministration. Surprisingly, a number of lung tumor‐nodules were observed from PNPs, Taxol or other control groups; in contrast, CNPs displayed few tumor nodules with a number of 0, demonstrating that CNPs completely inhibited lung metastasis (Figure 8D,E). These results were consistent with the findings for migration inhibition of cancer cells in vitro (Figure 4C,D).

Overall, potent therapeutic efficacy against breast cancer in mice was achieved due to effective intratumor and intracellular delivery of let‐7a with CNPs, as well as a synergistic effect between let‐7a and the chemotherapeutic, PTX, because the downregulation of KRAS protein by let‐7a enabled cancer cell sensitization to the chemotherapeutic agents.^24^

### 2.9 Safety Study

Severe toxicity to healthy organs consistently occurred following systemic injection of cationic vectors.^9^ After five intravenous injections at a PTX dose of 10 mg kg^−1^ or at 10 mg kg^−1^ of CLG or 5 mg kg^−1^ PEI into BALB/c mice, the main organs were collected and then subjected to H&E analysis at day 16. The injection of Taxol or PEI led to significant lymphocytic infiltration, microgranulation, and degenerative necrosis of hepatocytes and splenocytes compared with the saline‐treated mice in the H&E stain analysis, while no damage to these normal organs was observed following injection of PNPs or CNPs (Figure S5A, Supporting Information). Furthermore, the tolerability of cationic vectors without drug‐containing, CLG and PEI, was also evaluated by CD68 analysis after four injections. Treatment with cationic PEI or Taxol induced a profound inflammatory reaction in these organs in comparison with saline administration. Critically, little inflammation occurred in response to the injection of CLG, PNPs or CNPs (Figures S5B and S6, Supporting Information). In particular, liver toxicity stemming from the cationic polymers is an outstanding concern for gene delivery.^25^ Here, PNPs or CNPs did not induce damage to liver post‐treatment, probably owing to their escape from the liver over time without deposition at 24 h postinjection (Figure 6C). These data suggested that CLG, PNPs or CNPs were highly biocompatible.

## 3 Discussion

Rod‐shaped PNPs enabled safe and efficient miRNA delivery to target tissues and cancer cells. Indeed, commonly employed cationic vectors, such as PEI or PAMAM, which are capable of condensing genes via electrostatic interactions and, as a result, forming polyplexes, provide high transfection efficiency at the cellular level in vitro. However, their poor performances in vivo and, in particular, toxicity hinder their clinic applications. Herein, we present a novel gene delivery system mediated by PNPs. Unlike other cationic vectors, these vectors induced little toxicity to cells, as well as normal organs (heart, live, kidney, lung, and spleen), thereby suggesting their better biocompatibility. Conventional vectors render gene escape from lysosomes to cytoplasm of target cells via a mechanism of “proton sponge effect,”^26^ yet this escape efficiency is extremely low at only 1–2%.^12^ Moreover, this process results in rupture of the lysosome membrane and, in turn, induces cytotoxicity.^27^ This phenomenon can be partly explained that because of bypassing lysosomes following cellular uptake CNPs have markedly lower toxicity to cancer cells compared with PEI/let‐7a complexes (Figure 5H). Most importantly, CNPs entered cells via the caveolar route without entrapment in digestive lysosomes (≈pH 4.5) (Figure 2A–C), demonstrating the potential to protect miRNAs from enzymatic and acidic degradation and thus improving cytosolic localization of the gene. By this caveolar endocytic route, CNPs rendered efficient let‐7a delivery to cancer cells in target tissue, as evidenced by a reduction of KRAS expression by 50% in cancer cells postincubation with CNPs for 4 h and increase in let‐7a expression by 1.5‐fold and decline of KRAS expression by threefold in tumors treated with CNPs. Indeed, the efficient intratumoral and intracellular delivery of let‐7a was confirmed by the studies of antitumor activities in vivo and biodistribution. Furthermore, by contrast, the present CNPs with a positive charge and gene coating were distinguished from previously reported rod‐like drug particles (≈160 nm in length) with a negative charge,^16^ nevertheless, both of these two types of drug particles obtained cellular entry via the caveolar route, indicating that the change in surface of this type of particles would not alter their internalization pathway. Consequently, we believe that PNPs may also provide a valuable approach for efficient delivery of other proteins and peptides in vitro and in vivo. Further work to assess this hypothesis is currently underway.

Following systemic injection, rapid blood removal, nonspecific biodistribution, and poor tissue penetration are other main barriers against efficient gene delivery.^28^ In the pharmacokinetic study, we found that CNPs had a dramatically prolonged time of blood circulation, which improved their tumor accumulation via the EPR effect. Interestingly, CNPs exhibited most accumulation in the tumor, with profound tumor penetration compared with other organs, especially two reticuloendothelial system (RES) organs, liver and lung; at 24 h after dosing, CNPs distributed in the tumor were not removed, while their accumulation in liver or lung decreased significantly. These features of CNPs might be associated with their cellular uptake via the caveolar route, which resulted in the deposition of nanoparticles in RES by transcytosis and re‐entry into the blood circulation.^29^ These results demonstrated that CNPs were able to selectively accumulate in the tumor and therefore have potential to overcome these barriers for successful gene delivery in vivo.

Rod‐shaped PNPs‐mediated gene delivery allowed for a new combination approach of a chemotherapeutic agent and miRNA for cancer therapy. As reported previously, combined use of a small molecule drugs and genes with vectors, including liposomes, polymeric micelles, and inorganic nanoparticles, is a promising approach for disease therapy. However, the drug‐loading of these vectors has been low, not greater than 10%,^30^ thereby discounting therapeutic outcomes. Here, the drug‐loading in CNPs reached up to 500%, and thus this combined strategy has the potential to overcome this limitation of commonly utilized vectors. Conversely, in combination cancer treatment, the sequence and timing of individual drugs always affects the therapeutic effects,^31^ as a result, in the case of combined use of cytotoxic drug and miRNA, only when the release of the drug from the vectors after internalization lagged was it possible to obtain a maximum synergistic effect because regulation of target proteins by miRNA was of time consumption.^32^ However, conventional vectors for codelivery simultaneously released their drugs upon entry into cells because of their entrapment in lysosomes and subsequent decomposition. In this study, CNPs with insoluble drug particles as vectors for codelivery of a drug and miRNA may release the drug in a slow pattern after miRNA disassociation in cells, naturally inducing a sequence‐ and time‐related manner of administration and enhancing combined therapy. Truly, CNPs, a combination of drug particles of PTX and let‐7a, displayed a potently synergistic effect against cancer in terms of ≈100% tumor growth inhibition, an efficient apoptotic effect against cancer cells, ≈100% inhibition of lung metastasis, and other effects compared with single treatment with PNPs or Taxol. We also demonstrated that the combination of PTX with let‐7a is a potent approach for the treatment of highly metastatic cancer due to their complementary mechanisms. Additionally, downregulation of KRAS protein in cancer cells by let‐7a leads to increased expression of Cave‐1 on the cell surface^33^ and thus facilitates cellular uptake of the rod‐shape CNPs and improves treatment outcomes.

## 4 Conclusions

This study establishes that rod‐shape active particles that enter cells without entrapment in the digestive lysosomes are potent vectors for intracellular delivery of miRNAs. To the best of our knowledge, few reports have demonstrated that active nanosized drug particles can be used as vectors for drug delivery, in particular gene delivery; hence, our work offers a new avenue for drug delivery. We also believe that this miRNA‐replacement therapeutic strategy can be employed to efficiently deliver other proteins and peptides in vitro and in vivo for other diseases. It is worth noting that this delivery approach allows for easy combinations of drugs with miRNAs or other proteins and consequently has great potential to improve the therapeutic outcomes of drugs. Additionally, no complicated process is involved in the preparation of this nanoplatform, enabling scale‐up and supporting early clinical evaluations. Taken together, a new nanoplatform based on rod‐shape active particles, which obtain cellular entry by bypassing the lysosomal route, is presented for efficient and safe delivery of miRNAs. We believe that the nanoplatform reported herein is also adapted for protein delivery. Further work regarding the intracellular delivery of human Caspase‐3 to cancer cells is currently underway.

## 5 Experimental Section

Please refer to the Supporting Information for the Experimental Section. The animals received care in compliance with the Principles of Laboratory Animal Care and the Guide for the Care and Use of Laboratory Animals. Animal experiments followed a protocol approved by the China Pharmaceutical University Institutional Animal Care and Use Committee.

## Conflict of Interest

The authors declare no conflict of interest.

## Supporting information

Supplementary
