# Supplementary material for: Rod‐Shaped Active Drug Particles Enable Efficient and Safe Gene Delivery
Source: Adv Sci (Weinh). 2017 Sep 5;4(11):1700324. doi: 10.1002/advs.201700324 (PMC5700648; doi:10.1002/advs.201700324)
Supplement: Supplementary file 1 — Supplementary [file ADVS-4-na-s001.pdf]

Copyright WILEY-VCH Verlag GmbH & Co. KGaA, 69469 Weinheim, Germany, 2017.

Supporting Information

for *Adv. Sci.*, DOI: 10.1002/advs.201700324

Rod-Shaped Active Drug Particles Enable Efficient and Safe Gene Delivery

Xiaofei Xin, Xue Pei, Xin Yang, Yaqi Lv, Li Zhang, Wei He, and Lifang Yin**

Supporting Information

Rod-shaped active drug particles enable efficient and safe gene delivery

Xiaofei Xin [a], Xue Pei [a], Xin Yang [a], Yaqi Lv [a], Li Zhang [a], Wei He [a, *], Lifang Yin [a, b, *]

X.F. Xin, P. Xue, X. Yang, Y.Q. Lv, L. Zhang, Prof. W. He *, and Prof. L.F. Yin *

[a] Department of Pharmaceutics, School of Pharmacy, China Pharmaceutical University, Nanjing, 210009, PR China

[b] Key Laboratory of Druggability of Biopharmaceutics, China Pharmaceutical University, Nanjing, 210009, PR China

Corresponding authors to Wei He or Lifang Yin

Tel.: +86 2583271018; fax: +86 2583271018.

E-mail: weihe@cpu.edu.cn, lifangyin_@163.com

1. Experimental section

1.1. Synthesis of cationic β-lactoglobulin (CLG)

CLG was synthesized by conjugation of ethylenediamine to beta-lactoglobulin (β-LG). Ethylenediamine solution (0.9 M, 250 mL, pH = 4.75) was slowly added to 2 mL of β-LG solution (15%, m/v) with continuous stirring, following by addition of 70 mg of 1-Ethyl-3-(3-dimethylaminopropyl) carbodiimide (EDCI). After incubation at room temperature for 2 h, the reaction was quenched by addition of 200 μL acetate buffer (4 M, pH = 4.75), extensively dialyzed against water and freeze-dried in sequence.

1.2. Preparation and characterization of PNPs and CNPs

The PNPs were prepared via an antisolvent-precipitation method as previously reported.[1] In brief, 10 mL of the CLG solution (1 mg/mL) and 1 mL of acetone containing 20 mg of PTX were used as the aqueous phase and organic phase, respectively. After being precooled to below 4 °C in an ice-water bath, the organic phase was placed into the aqueous phase under stirring conditions. Subsequently, the samples were immediately treated with an ultrasonic probe (20-25 kHz, Scientz Biotechnology Co., Ltd., Ningbo, China) at 400 W for 15 min. Finally, the samples were evaporated under reduced pressure to remove the residual acetone. To prepare CNPs, the desired amounts of lethal-7a (let-7a) were mixed with equal volume of PNPs, and then the resultant mixture was vortexed gently for 30 s followed by incubation at room temperature for 30 min. Scrambled let-7a/PNPs complexes (SNPs) were prepared by the same method as described above. Using a similar procedure, fluorescein isothiocyanate isomer I-labeled CNPs (FITC-CNPs) were prepared by dissolving fluorescein isothiocyanate isomer I (FITC) with PTX together as the organic phase prior to mixing with CLG protein. Cy5-labeled CNPs (Cy5-CNPs) and dual-labeled CNPs were fabricated by assembling Cy5-let-7a on PNPs and FITC-CNPs, respectively.

The particle size, particle size distribution and zeta-potential were determined

using a 90Plus Particle Size Analyzer (Brookhaven Instruments, Holtsville, NY) at 25 °C according to the dynamic light scattering (DLS) principle. The size distribution was indicated as the polydispersity index (PDI). The raw data were collected over 5 min at an angle of 90° and expressed as intensity-weighted Gaussian distributions. The samples were diluted 50- to 100-fold in advance of examination.

The examination of the nanoparticle's shape was carried out on a JEM-1230 transmission electron microscope (TEM, Tokyo, Japan) under an acceleration voltage of 200 kV. After a 200-fold dilution, one drop of sample was placed on a copper mesh, and then the excess sample material was removed with filter paper. Afterwards, one drop of 2% (w/w) phosphotungstic acid was added to the copper and allowed to stain for 30 s. Finally, after removing the extra phosphotungstic acid, the mesh was dried at 25 °C for 5 min.

1.3. Circular dichroism (CD) spectroscopy and fluorescence measurements

The emission spectrum from 300-500 nm was scanned at an excitation wavelength of 295 nm using a fluorescence spectrometer (SHIMADZU RF-5301PC, Japan). The samples for analysis were CLG and PNPs with different drug-loadings. CD spectra were recorded using a J-810 spectrometer (Tokyo, Japan) equipped with a temperature-controlling unit and a quartz cuvette, and the ellipticity was expressed in millidegrees. The parameters for determination were described as follows: bandwidth, 1 nm; response,1 s; wavelength range, 250–190 nm; scanning speed, 100 nm/min; cell length, 0.1 cm; temperature, 25 °C; and protein concentration 0.1 mg/mL.

1.4. RNA binding ability and stability assay

The RNA binding ability and RNA stability against degradation by RNase and serum were tested by agarose gel electrophoresis,[2] for which the gels were prepared with 2% agarose in ethylenediaminetetraacetic acid (EDTA) buffer containing 0.5 μg/mL GelRED (Biotium, USA).

Briefly, CNPs were prepared at various weight ratios of CLG/let-7a. The samples

were incubated with 5× loading buffer containing GelRed (Generay Biotechnology, China) for 30 min. and then 10% glycerine and 5 μL of 2% sodium dodecyl sulfate (SDS) were added to the mixture. Gel electrophoresis was performed at 110 V for 30 min. Finally, the gel was photographed using Bio-Rad high-sensitivity chemiluminescence imaging system (Chemidoc XRS+, USA).

To assess the protection for let-7a in CNPs against degradation by RNase A, naked let-7a (0.5 μg) or CNPs were cultured with 10 μg/mL RNase A at 37 °C. At predetermined time points, the resultant samples were stopped by incubation with 1% SDS for 5 min at 60 °C followed by addition of 2% SDS to displace miRNAs from CNPs. The samples were assayed by agarose gel electrophoresis as described above.

To evaluate the serum stability of let-7a in CNPs, CNPs were cultured in 10% FBS solution at 37 °C for different times. Then, the samples were removed, processed according to the procedure described above and finally assayed by agarose gel electrophoresis.

1.5. *In vitro* drug release

The drug release from Taxol, PNPs and CNPs was tested using a dialysis method performed in an incubator (SHA-C, Jintan, China) with a shaking speed of 100 rpm/min at 37 ± 1 °C. The molecular weight cutoff of the dialysis bag was 3500 Da, and the release medium was phosphate-buffered saline (PBS) with three different pH values (pH 7.4, 6.8 and 5.0). At predetermined intervals, samples were withdrawn from outside the dialysis bag and replaced with an equal volume of corresponding fresh PBS. After filtration through a 0.2-μm membrane filter, the PTX content in these samples was detected by high performance liquid chromatography (HPLC) as depicted in a previous report.[3]

1.6. Caveolae-mediated cellular uptake

The 4T1 and Caco-2 cells were seeded on 12-well plates at a density of 2×10^5 cells/well and cultured for 48 h at 37 °C, respectively. After pretreatment with

caveolae inhibitors, nystatin (10 μM) or methyl-β-cyclodextrin (M-CD, 2.5 mM), for 0.5 h at 37 °C, the cells were incubated with dual-labeled CNPs in serum-free culture medium at 37 °C for 4 h. At the end of the experiment, the cells were washed three times with PBS and collected by trypsinization to assess the fluorescence intensity by flow cytometry (FCM, BD FACSCalibur, USA).

1.7. Co-localization of CNPs with caveolae

For the location detection of CNPs in caveolae, Caco-2 cells were incubated with the CNPs at 100 nM Cy5-let-7a in DMEM for 4 h at 37 °C. Subsequently, the cells were sequentially washed, fixed with 4% (w/w) paraformaldehyde for 10 min, permeabilized in 0.1% Triton X-100 for 5 min, and blocked in blocking buffer (1% bovine serum albumin/10% normal goat serum/0.3 M glycine in 0.1% PBS-Tween) for 15 min. The cells were then incubated with Caveolae Marker (Alexa Fluor® 488) at a working dilution of 1 in 50 for 2 h at room temperature. After washing, the co-localization was examined by confocal laser scanning microscopy (CLSM, LSM700, Carl Zeiss, Germany).

For the co-localization of CNPs with actin, a similar procedure was conducted as described above. Briefly, the cells were cultured with Cy5-CNPs at 100 nM Cy5-let-7a for 4 h at 37 °C. The cells were then rinsed and fixed with 4% (w/w) paraformaldehyde (10 min), permeabilized in 0.5% Triton X-100 for 5 min, and incubated with 200 nM FITC phalloidine in PBS containing 1% bovine serum albumin for 30 min at room temperature.

To study the co-localization of PTX-Ns with cholera toxin subunit B (CTB), the cells were co-incubated with Alexa Fluor® 488-CTB at 20 μg/mL and Cy5-CNPs at 100 nM Cy5-let-7a in DMEM for 3 h at 37 °C. The cells were then rinsed and observed by CLSM (LSM700, Carl Zeiss, Germany).

1.8. Cellular trafficking and time lapse fluorescence imaging

The 4T1 and Caco-2 cells were seeded on 30-mm glass-bottomed cell culture dishes

at a density of 1×10^5 cells/dish and cultured for 48 h. The cells were then cultured with FITC-CNPs at 100 μg/mL FITC or Cy5-CNPs at 100 nM Cy5-let-7a in serum-free culture medium for 4 h at 37 °C, following by addition of 1 mL of Lyso-tracker red or Lyso-tracker green and incubation for 1 h at 37 °C. The cells were examined with CLSM after three rinses with PBS.

To confirm the co delivery of PNPs and let-7a to cells, cellular uptake of dual-labeled CNPs was performed using a method similar to that described above, except that the lysosomes were not marked by staining with Lyso-tracker red or green.

For time-lapse live cell imaging, 4T1 cells at a density of 1×10^5 were cultured in the dish for 48 h prior to use. After incubation with Cy5-CNPs at 100 nM Cy5-let-7a, the cells were placed in the live cell imaging chamber with 5% CO_2 at 37 °C on the CLSM (LSM700, Carl Zeiss, Germany). A series of images were obtained at 30-s intervals over a period of 30 min.

1.9. Transcytosis

The *in vitro* transcellular model was prepared as described in a previous report.[4] In brief, Caco-2 cells at a density of 2×10^5 cells /well were cultured on a 12-well polycarbonate Transwell filter with a pore size of 3 mm and diameter of 12 mm (Costar, Corning, NY) for 7 days. Culture medium in both apical and basolateral chambers was renewed every 2 days during the culture period. The integrity of the cell monolayer was confirmed by determining the transepithelial electrical resistance (TEER), and the cell monolayer with a TEER value greater than 500 Ω cm^2 was chosen for further analysis.

The cell monolayers on porous Transwell insert membranes were incubated with Cy5-CNPs at 100 nM Cy5-let-7a and dual-labeled CNPs at FITC 10 μg/mL at 37 °C for a predetermined duration. Subsequently, the polycarbonate membranes covered with the cell monolayers were isolated from the Transwell insert and washed with cold PBS, fixed with 4% paraformaldehyde for 10 min, and stained to detect F-actin and nuclei with FITC-phalloidine and 4’,6-diamidino-2-phenylindole (DAPI) for 30

min, respectively. Finally, the membranes were examined by CLSM (LSM700, Carl Zeiss, Germany) in X-Y-Z scanning mode after placement and sealing on glass slides. At designated time points, the media in both apical and basolateral chambers were collected and subjected to size-distribution analysis by DLS.

1.10. Penetration into multicellular spheroids

A multicellular spheroid model was employed to determine the ability of CNPs to penetrate the tumor tissue.[3] In brief, 4T1 cells with a density of 2×10^3 cells/well were cultured in a 96-well plate for 7 days at 37 °C. Then, the spheroids were incubated with dual-labeled CNPs with 10 µg/mL FITC or 100 nM Cy5-let-7a at 37 °C. At predetermined time intervals, the location of CNPs in the tumor spheroids was observed using CLSM (LSM700, Carl Zeiss, Germany). The penetration ratio (%) was also calculated using the ratio of the penetration depth from the surface to center of the spheroid diameter.

1.11. Cytotoxicity and apoptosis

Cell viability was assessed using 3-(4,5-dimethylthiazol-2yl)-2,5-diphenyltetrazoliumbromide (MTT) and lactate dehydrogenase (LDH) assays. The 4T1, A549, and Caco-2 cells were seeded on 96-well plates at a density of 5000 cells/well and cultured for 48 h to allow them to adhere. The culture media were removed and then replaced with 200 μL of RPMI 1640 supplemented with 10 % fetal bovine serum containing CLG, PEI, SDS, Taxol, naked let-7a, PNPs and CNPs for 48 h. The tested drug-concentrations were as follows: 25–400 nM for miRNA, 0.5–100 µg/mL for PTX, 0.25–500 µg/mL for CLG, and 100 µg/mL for both PEI and SDS. After culturing for 48 h at 37 °C, 20 µL of MTT at a concentration of 5 mg/mL was placed in each well and cultured for another 4 h. After removing the medium, 200 µL of dimethyl sulfoxide (DMSO) was added to each well to dissolve the formazan crystals, and the absorbance at 570 nm was determined with a Microplate Reader (Multiskan FC, USA).

LDH is a soluble enzyme that resides in the cytosol and is released into the

culture medium once the cell membrane is broken. The LDH assay is able to detect the amount of cytoplasmic LDH release into the medium as function of the membrane integrity. The cytotoxicity of CLG, PEI and SDS was further evaluated by the LDH assay. Briefly, the Caco-2 or 4T1 cells were incubated with CLG at concentrations from 0.25–500 μg/mL, 100 μg/mL PEI and 0.25% SDS for 48 h at 37 °C, respectively, whereas PBS was used as the negative control. Next, 120 μL of the sample solution was obtained from each well and placed in another multi-well plate, following by addition of 60 μL LDH reagent, incubation for 30 min at room temperature, and absorbance detection at 490 nm with a spectrophotometer (Multiskan FC, USA) with LDH release solution as a positive control.

Cell apoptosis was studied by the cell nuclear shape by DAPI staining and Annexin V-FITC/PI apoptosis detection kits (Shanghai Yeasen Biotechnology Co., Ltd., Shanghai, China), respectively. In brief, the cells were treated with Taxol, naked let-7a, CLG, PNPs or CNPs at a fixed PTX concentration of 10 μg/mL or 100 nM of let-7a for 48 h at 37 °C. The treated cells were sequentially rinsed three times with PBS, stained with DAPI at room temperature for 10 min, and examined using an Olympus IX53 fluorescence microscope (Japan). Cell apoptosis was assessed based on alterations in nuclear morphology in terms of chromatin condensation, fragmentation and apoptotic body formation. Determination of cell apoptosis was also performed with Annexin V-FITC/PI apoptosis detection kits and FCM. Cells were treated as described above, collected after incubation with the drug formulations, processed according to the manufacturer's protocol, and finally analyzed by FCM.

1.12. Migration of cancer cell inhibition

The migration assay was performed on transwell insert chambers (Corning, USA). The 4T1 cells were cultured at a density of 6×10^5 cells/well in 6-well plates for 48 h prior to use. The cells were incubated with Taxol, naked let-7a, CLG, PNPs and CNPs in 2 mL of culture media without serum for 4 h at 10 μg/mL PTX or 100 nM let-7a, followed by two washes with PBS and incubation in RPMI-1640 medium with 10%

FBS for an additional 24 h. The cells were then harvested and added to the upper chamber in serum-free medium, and 20% FBS in the lower chamber served as the chemoattractant. After incubation for 24 h at 37 °C, the cells in the upper chambers were collected via wiping with a cotton swab, whereas the cells that had migrated to the lower surface of filter were fixed in 4% paraformaldehyde for 10 min and stained with 0.1% crystal violet for 10 min. The marked cells were examined under a light microscope. Finally, the crystal violet was dissolved in acetic acid (33%, w/v), and the optical density (OD) ratio was detected at 570 nm using a microplate reader.

1.13. Transfection determination by quantitative real-time PCR and western blot assay

Quantitative real-time PCR (qRT-PCR) was used to assess the expression level of KRAS mRNA. Briefly, 4T1 cells at a density of 2×10^5 cells/well were seeded in 6-well culture plates for 48 h in advance. The cells were incubated with PBS, CLG (150 µg/mL), Taxol, PNPs, or CNPs, respectively, for 6 h at 37 °C, or with 10 µg/mL PTX or 100 nM let-7a. The media were then replaced with fresh media, followed by incubation for 48 h. Total RNA was then extracted with TRIzol reagent (Invitrogen, USA) according to the manufacturer's protocol, and qRT-PCR was performed on an ABI Step One Plus Real-time PCR system (Applied Biosystems, USA) equipped with SYBR® Green Real-time PCR Master Mix (TOYOBO, Japan). PCR reactions were conducted three times. The details of the procedure were as follows: initial denaturation: 95 °C for 5 min; 40 cycles: 15 s at 95 °C, 20 s at 60 °C, 40 s at 72 °C; and 1 cycle: 15 s at 95 °C, 1 min at 65 °C, 15 s at 95 °C. GAPDH was employed as an endogenous control to normalize expression.

For the western blot assay, the treated cells were lysed in cold lysis buffer followed by centrifugation at 10, 000 g for 10 min, and then, the supernatants were collected for protein determination with the BCA protein assay kit (KeyGEN Biotech., China) and protein separation by SDS-PAGE. Subsequently, the separated proteins were transferred onto a PVDF membrane (Millipore, USA), incubated with blocking

solution (5% fat-free dried milk) at room temperature for 1 h, treated with a monoclonal antibody against KRAS overnight at 4 °C, cultured with goat anti-rabbit secondary antibody for 1 h, and stained with an ECL chemiluminescence kit (KeyGEN Biotech., China). Finally, blotting images were obtained from a G: Box ChemiXR5 (Syngene, Cambridge, UK) using GAPDH as an internal reference for normalization.

1.14. Blood circulation study

The animals used in the experiments received care in compliance with the Principles of Laboratory Animal Care and the Guide for the Care and Use of Laboratory Animals. All the animal experiments were performed in accordance with the protocol approved by the China Pharmaceutical University Institutional Animal Care and Use Committee.

The *in vivo* pharmacokinetics of Taxol (free drug), PNPs and CNPs were investigated in rats. The rats were randomly divided into three groups (4 rats per group) and intravenously injected with Taxol, PNPs or CNPs, respectively, at a PTX dose of 10 mg/kg, according to the animal's body weight. At specific time intervals, 0.5-mL blood samples were collected from the orbit and then immediately centrifuged for 10 min at 5,000 g (Anke TGL-16G, Shanghai, China) to separate the plasma. The extraction of PTX from plasma and determination of the plasma concentration were performed as described in a previous report.[5] The pharmacokinetic parameters were calculated based on a statistical moment theory using the Microsoft Excel 2007 program.

1.15. *Ex vivo* imaging and biodistribution

The 4T1 tumor-bearing BALB/c mice were used in studies of biodistribution and antitumor activities. Briefly, 0.2 mL of suspended 4T1 cells with a density of 1×10^7 cells/mL was subcutaneously injected into the scapula region of the mice. The mice with a tumor volume of approximately $10 \times 10 \times 5$ mm^3 were selected for subsequent experiments.

The biodistribution of dual-labeled CNPs were analyzed in the 4T1 tumor-bearing mice. Eighteen mice were randomly divided into 3 groups with 6 mice each and then intravenously injected via the tail vein with 0.2 mL of dual-labeled CNPs, free FITC, or Cy5-let-7a at 750 μg/kg FITC or 1 mg/kg Cy5-let-7a according to the animal's body weight. At predetermined time points, the mice were killed to collect their major organs including their hearts, livers, spleens, lungs and kidneys, followed by imaging *ex vivo* to detect the fluorescence intensity using an *in-vivo* imaging system (In-Vivo FX PRO, Carestream, Canada). The excitation wavelengths were 470 nm and 630 nm for FITC and Cy5, respectively, and the emission wavelengths were 530 nm and 670 nm for FITC and Cy5, respectively.

To further investigate the accumulation of CNPs in the tumors, the tumors were excised from the mice at 2 h after administration of dual-labeled CNPs. After freezing, the tumors were cut into 6–8 μm sections with a microtome (Leica CM1860, Germany), stained with DAPI for 15 min, and finally observed by CLSM.

1.16. Therapeutic efficacy

The 4T1 tumor-bearing BALB/c mice were randomly divided into seven groups (12 mice/group): saline, CLG, naked let-7a, PEI/let-7a complexes, Taxol, PNPs and CNPs. The mice were intravenously injected via the tail vein with 0.2 mL of these formulations every 3 days for 5 times at a fixed PTX dose of 10 mg/kg and let-7a dose of 1 mg/kg, according to the animal's body weight. The tumor volume and body weight were determined at predetermined time intervals. The survival rate of animal was also monitored during treatment. On post-injection day 16, half of the mice in each group was killed to collect the tumor tissues and lungs. The isolated tumors were subjected to HE staining, TUNEL assay and Ki67 staining, respectively, and the lungs were fixed in Bouin's fixative for 24 h to examine the tumor burden in the lungs. The expressions level of KRAS mRNA and protein in tumor tissues were assessed by qRT-PCR and western blot, respectively. The other mice were subjected to the survival study until day 30, and the endpoint was detected as depicted in a previous

report.[6]

1.17. Safety study

Female BALB/c mice were randomly divided into groups (n = 6 for each group) that received an intravenous injection of saline, CLG, PEI, Taxol, naked let-7a, PEI/let-7a complexes, PNPs and CNPs via the tail vein at 1, 4, 7, 10 and 13 days. The administered doses of active or inactive ingredients in these formulations were the same as described for the study of antitumor efficacy. At 15 days post-administration, the mice were sacrificed to harvest the major tissues, following by preparation of 5-μm-thick tissue sections and H&E and CD68 immunohistochemical analysis.

1.18. Statistical analysis

One-way analysis of variance was performed to assess the statistical significance of the differences between samples. The results are expressed as the means ± standard deviation. Significant differences were set as $P < 0.05$.

References

[1] Y. Li, Z. Wu, W. He, C. Qin, J. Yao, J. Zhou, L. Yin, *Mol. Pharmaceut.* **2015**, *12*, 1485.

[2] L. Zhang, X. Yang, Y. Lv, X. Xin, C. Qin, X. Han, L. Yang, W. He, L. Yin, *Sci. Rep.* **2017**, *7*, 46186.

[3] W. He, X. Xin, Y. Li, X. Han, C. Qin, L. Yin, *Part. Part. Syst. Charact.* **2017**, 34, 6, 1600371.

[4] S. Zhao, W. Dai, B. He, J. Wang, Z. He, X. Zhang, Q. Zhang, *J. Control. Release* **2012**, *158*, 413.

[5] Z. Jin, Y. Lv, H. Cao, J. Yao, J. Zhou, W. He, L. Yin, *Sci. Rep.* **2016**, *6*, 27559.

[6] C. P. Hollis, H. L. Weiss, M. Leggas, B. M. Evers, R. A. Gemeinhart, T. Li, *J. Control. Release* **2013**, *172*, 12.

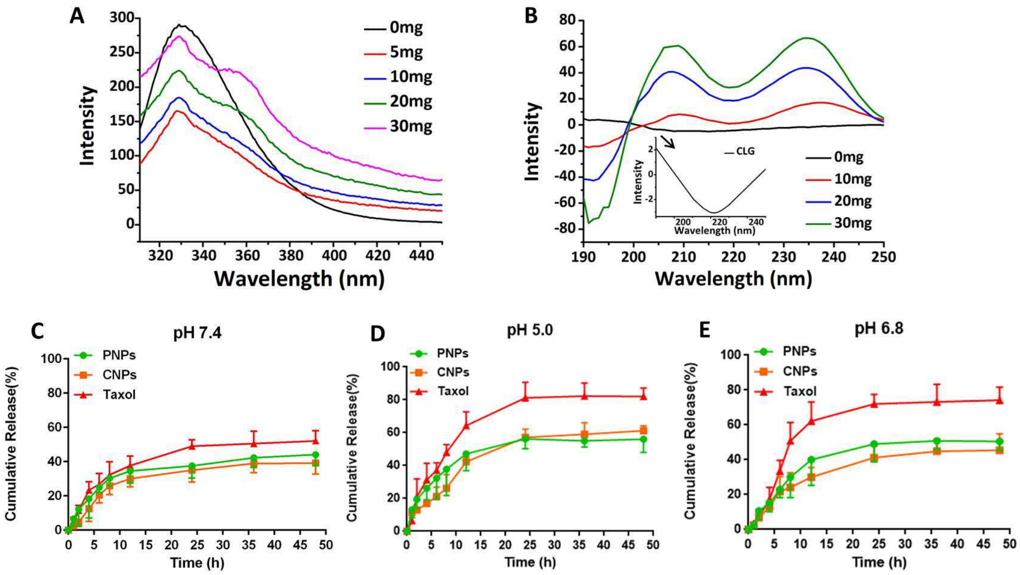

Figure S1. Interaction study between PTX particles and CLG. (**A**) Fluorescence emission and (**B**) far UV-CD spectrum of PNPs with 100%–300% drug-loadings (10 mg–30 mg in formulations). (**C–E**) *In vitro* release profile of PTX from Taxol, PNPs and CNPs in PBS solutions containing 1 M sodium salicylate at 37 °C for 48 h (C, pH 7.4; D, pH 5.0; E, pH 6.8).

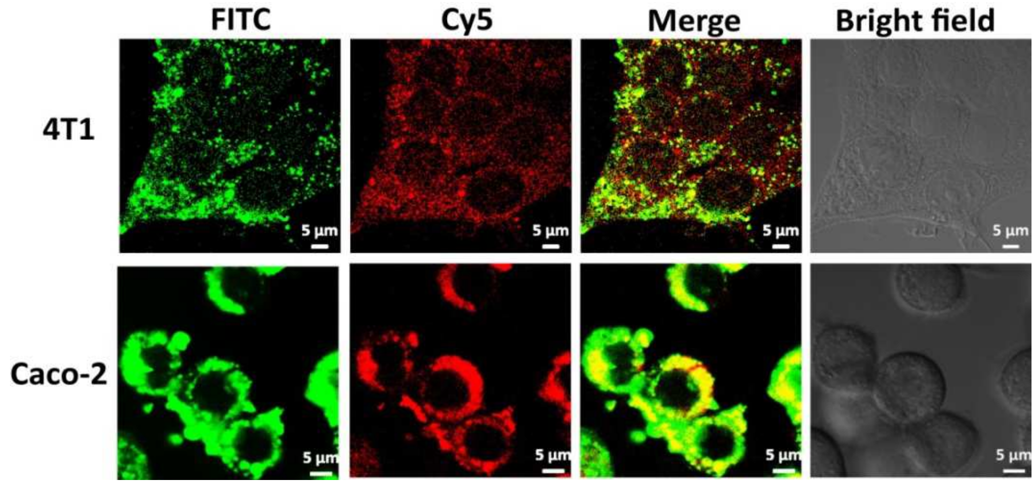

Figure S2. Location of the dual-labeled CNPs (10 μg/mL FITC and 100 nM let-7a) in 4T1 or Caco-2 cells by CLSM at 37 °C for 4 h, respectively. Yellow spots indicate the co-delivery of PTX and let-7a.

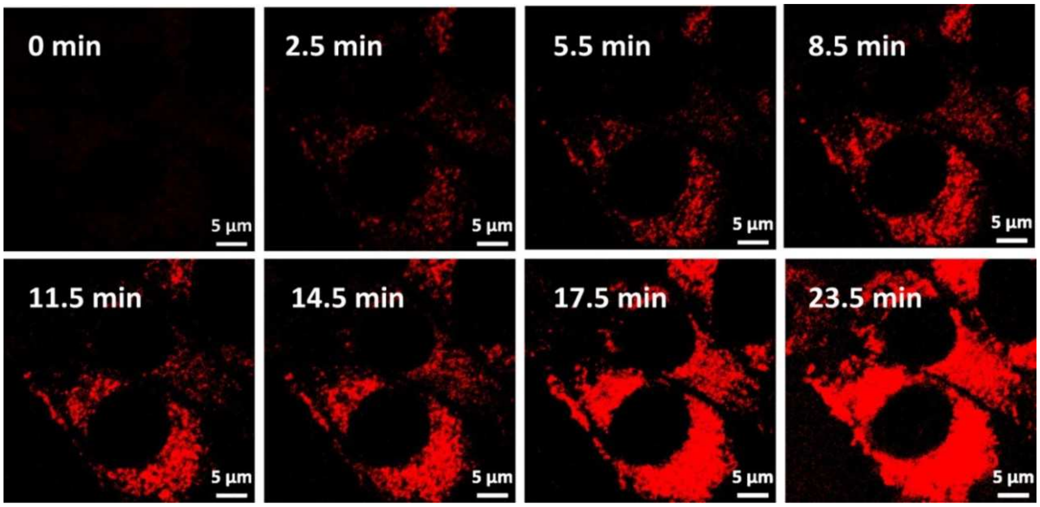

Figure S3. Live cell imaging of Cy5-CNPs (red, 100 nM let-7a) delivery into the cytosol of 4T1 cells at 37 °C.

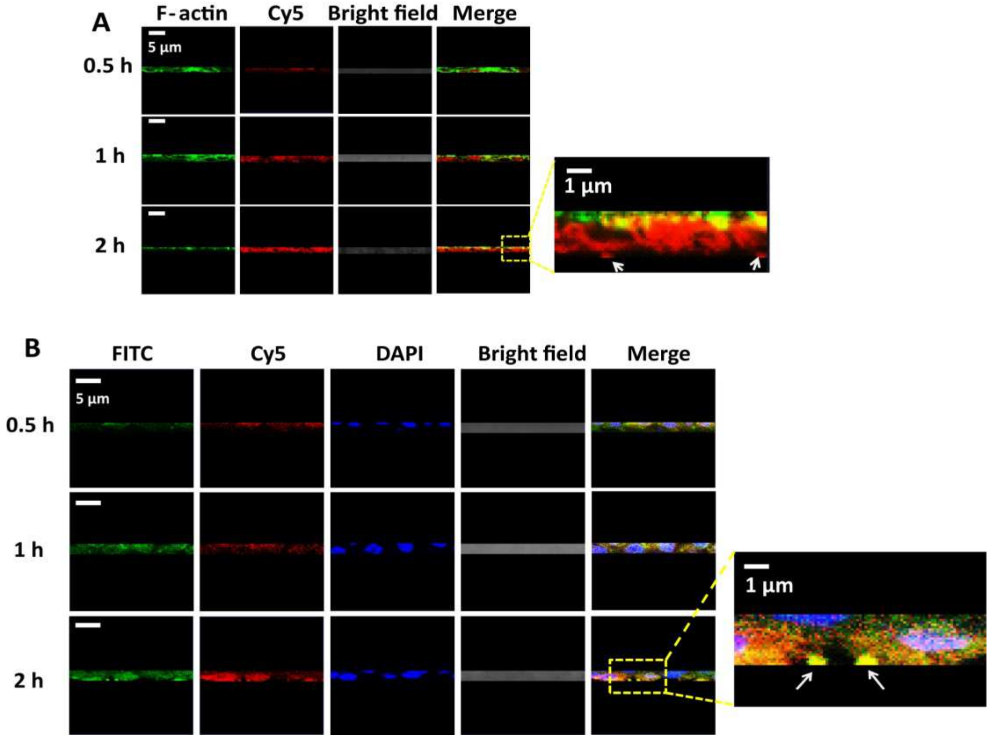

Figure S4. Transcytosis of CNPs across the Caco-2 cell monolayer. The X-Z vertical confocal micrographs of the Caco-2 cell monolayer after incubation with (**A**) Cy5-CNPs (100 nM) or (**B**) dual-labeled CNPs (FITC 10 μg/mL, Cy5-let-7a 100 nM) at 37 °C for 0.5 h, 1 h and 2 h, respectively. Cy5-CNPs (100 nM) were co-localized with FITC-labeled actin (F-actin, green). Cells were stained with DAPI to mark cell nuclei (blue). White arrows indicate the CNPs crossed the Caco-2 cell monolayer.

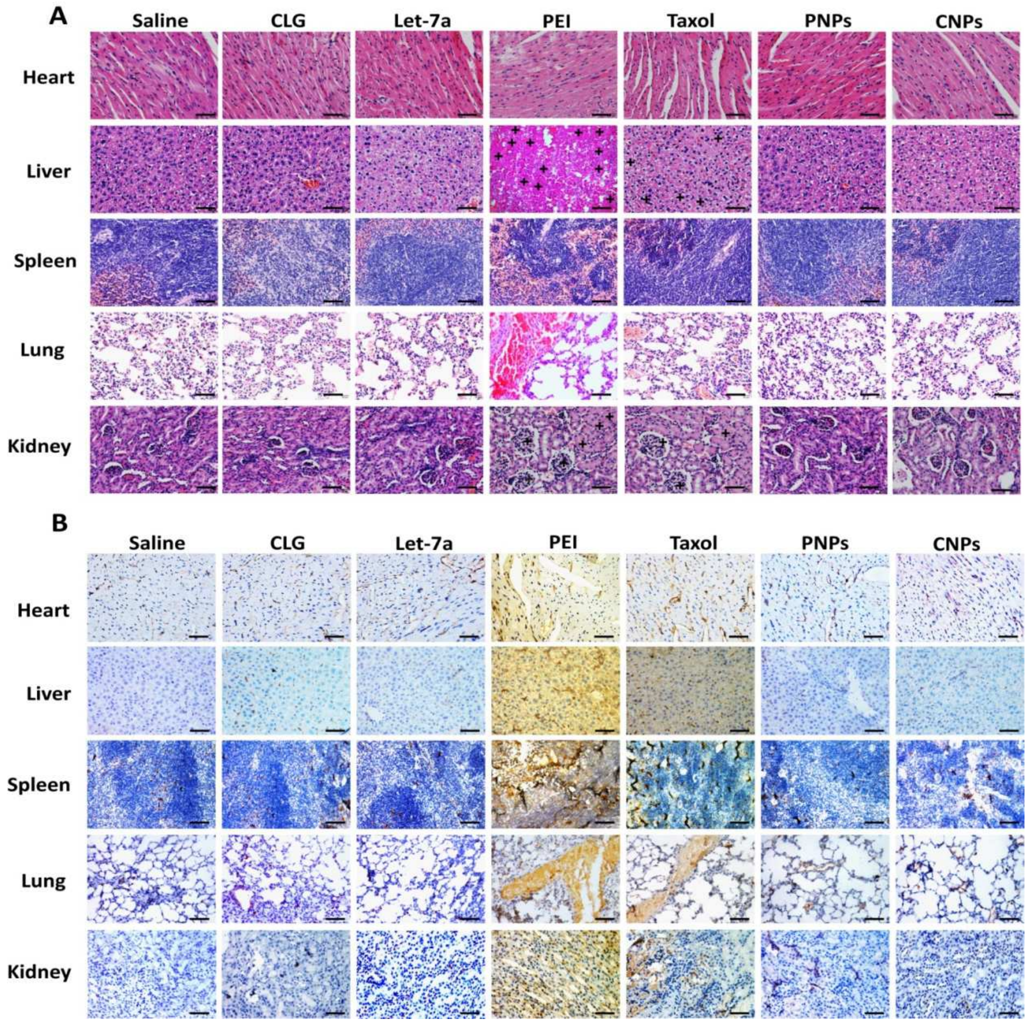

Figure S5. (**A**) Representative normal tissue histology after treatment with saline, CLG, naked let-7a, PEI, Taxol, PNPs and CNPs in mice. Saline, CLG, Taxol, PNPs and CNPs were administered to 4T1 tumor-bearing mice via tail vein injections every 3 days at a PTX dose of 10 mg/kg and let-7a dose of 1 mg/kg, according to the animal's body weight. The tumor tissues, liver, lung, spleen, heart and kidney were collected at day 16. Nuclei are stained blue while extracellular matrix and cytoplasm are stained red in the H&E analysis. "+" represents the positive area. The scale bar is 20 μm. (**B**) CD68 analysis of normal tissues after treatment with saline, CLG, naked let-7a, PEI, Taxol, PNPs, and CNPs in mice. Nuclei are stained blue, and the brown-stained cells represent positive cells. The scale bar is 20 μm.

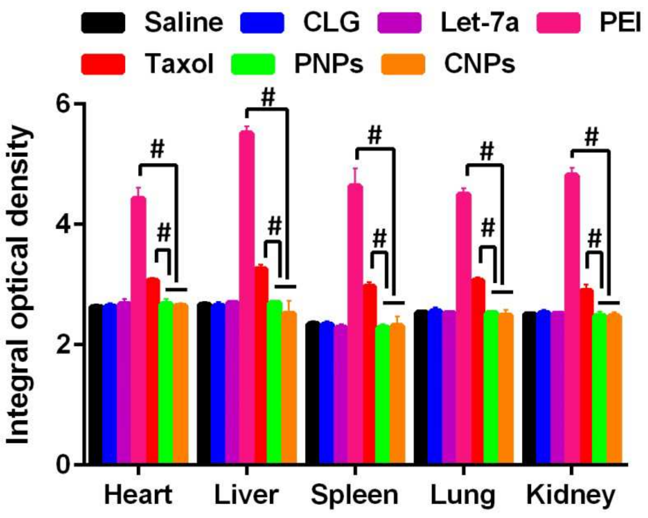

Figure S6. CD68 quantitative analysis of normal tissues after treatment with saline, CLG, naked let-7a, PEI, Taxol, PNPs, and CNPs in mice (n = 5, $^{\#}P < 0.001$).
